# Chronic kidney disease and risk of bloodstream infections and sepsis: a 17-year follow-up of the population-based Trøndelag Health Study in Norway

**DOI:** 10.1007/s15010-024-02265-2

**Published:** 2024-04-29

**Authors:** Kristin Vardheim Liyanarachi, Randi Marie Mohus, Tormod Rogne, Lise Tuset Gustad, Bjørn Olav Åsvold, Solfrid Romundstad, Erik Solligård, Stein Hallan, Jan Kristian Damås

**Affiliations:** 1 Mid-Norway Center for Sepsis Research, Department of Circulation and Medical Imaging, NTNU, Norwegian University of Science and Technology, Trondheim, Norway; 2 Department of Infectious Diseases, St. Olavs Hospital, Trondheim University Hospital, Trondheim, Norway; 3 Clinic of Anaesthesia and Intensive Care, St. Olavs Hospital, Trondheim University Hospital, Trondheim, Norway; 4 Yale Department of Chronic Disease Epidemiology and Center for Perinatal, Pediatric and Environmental Epidemiology, Yale School of Public Health, New Haven, CT USA; 5 Faculty of Nursing and Health Sciences, Nord University, Levanger, Norway; 6 Department of Internal Medicine, Levanger Hospital, Nord-Trøndelag Hospital Trust, Levanger, Norway; 7 K.G. Jebsen Center for Genetic Epidemiology, Department of Public Health and Nursing, NTNU, Norwegian University of Science and Technology, Trondheim, Norway; 8 Department of Endocrinology, Clinic of Medicine, St. Olavs Hospital, Trondheim University Hospital, Trondheim, Norway; 9 Department of Public Health and Nursing, HUNT Research Center, NTNU, Norwegian University of Science and Technology, Levanger, Norway; 10 Department of Clinical and Molecular Medicine, NTNU, Norwegian University of Science and Technology, Trondheim, Norway; 11 Helse Møre Og Romsdal Hospital Trust, Ålesund, Norway; 12 Department of Nephrology, St Olavs Hospital, Trondheim University Hospital, Trondheim, Norway; 13 Centre of Molecular Inflammation Research, Department of Clinical and Molecular Medicine, NTNU, Norwegian University of Science and Technology, Trondheim, Norway

**Keywords:** Sepsis, Chronic kidney failure, Blood-borne infections, Epidemiology, Sepsis epidemiology

## Abstract

**Purpose:**

Bloodstream infections (BSI) and sepsis are important causes of hospitalization, loss of health, and death globally. Targetable risk factors need to be identified to improve prevention and treatment. In this study, we aimed to evaluate the association of chronic kidney disease (CKD) and risk of and mortality from BSI and sepsis in the general population during a 22-year period.

**Methods:**

We conducted a prospective cohort study among participants in the population-based Norwegian HUNT Study, where 68,438 participated. The median follow-up time was 17.4 years. The exposures were estimated glomerular filtration rate (eGFR) and albumin–creatinine ratio (ACR) in urine. The outcomes were hazard ratios (HR) of hospital admission or death due to BSI or sepsis. The associations were adjusted for age, sex, diabetes, obesity, systolic blood pressure, smoking status, and cardiovascular disease.

**Results:**

Participants with eGFR < 30 ml/min/1.73^2^ had HR 3.35 for BSI (95% confidence intervals (CI) 2.12–5.3) and HR 2.94 for sepsis (95% CI 1.82–4.8) compared to normal eGFR (≥ 90 ml/min/1.73^2^). HRs of death from BSI and sepsis were 4.2 (95% CI 1.71–10.4) and 4.1 (95% CI 1.88–8.9), respectively. Participants with severely increased albuminuria (ACR > 30 mg/mmol) had HR 3.60 for BSI (95% CI 2.30–5.6) and 3.14 for sepsis (95% CI 1.94–5.1) compared to normal albumin excretion (ACR < 3 mg/mmol). HRs of death were 2.67 (95% CI 0.82–8.7) and 2.16 (95% CI 0.78–6.0), respectively.

**Conclusion:**

In this large population-based cohort study, CKD was clearly associated with an increased risk of BSI and sepsis and related death.

**Supplementary Information:**

The online version contains supplementary material available at 10.1007/s15010-024-02265-2.

## Introduction

Severe bacterial infection leading to sepsis is an important cause of hospitalization and loss of health worldwide [1, 2]. While the case fatality rate of sepsis is decreasing, the incidence is rising and will further rise as our population gets older [1, 3, 4]. To reduce the disease burden of sepsis and ensure proper disease risk stratification, it is important to identify targetable risk factors.

Previous epidemiological studies have identified chronic kidney disease (CKD), defined as abnormalities in the kidney structure or function, as a potential risk factor for sepsis. However, these previous studies are limited by evaluating subjects not representative of the general population [5–7], using a variety of and therefore imprecise definitions of CKD and sepsis [5, 6, 8–11], and having a short follow-up time [8, 10]. In particular, the evidence on the relationship between proteinuria and infection incidence is weak.

To address these issues, we aimed to evaluate the association of CKD, defined by both estimated glomerular filtration rate (eGFR) < 60 ml/min/1.73m^2^ and albuminuria (urinary albumin–creatinine ratio (ACR) ≥ 3 mg/mmol [12, 13], with the risk of and mortality from sepsis and bloodstream infections (BSI). We used the International Classification of Disease (ICD) codes and blood culture results to identify participants with sepsis and BSI in a 17-year follow-up in the population-based Trøndelag Health (HUNT) study.

## Materials and methods

### Description of the study cohort

We used baseline data from the second and third surveys of the Trøndelag Health Study, HUNT2 (1995–1997) and HUNT3 (2006–2008), in which a total of 79,393 subjects participated (69.5% and 54.1% of the invited population for HUNT2 and HUNT3, respectively). Most of the participants (72% of the women and 69% of the men) in HUNT2 also participated in HUNT3. The population is considered generally representative of the Norwegian population, and demographic descriptions and characteristics of the HUNT surveys have been published previously [14, 15]. The participants completed questionnaires covering a wide range of health-related topics, underwent clinical examination and blood collection, and were then followed from the day of first inclusion until February 2017. The follow-up time was up to 22.8 years (median 17.4 years).

### Exposures

We used the creatinine-based European Kidney Function Consortium (EKFC) equation to estimate GFR since this has improved accuracy in European cohorts [16]. eGFR was then categorized according to the Kidney Disease: Improving Global Outcomes (KDIGO) clinical guidelines (≥ 90, 60–89, 45–59, 30–44, 15–29, and < 15 ml/min/1.73m^2^) [17]. The two lowest categories were classed together.

The ACR was used as an expression for urine albumin excretion. Participants with self-reported diabetes or self-reported use of antihypertensive drugs, in addition to a random sample of 5% of the whole study population, were asked to deliver three urine samples using prepaid envelopes and standardized receptacles. Participants with self-reported urinary tract infection, visible hematuria, or menstruation while collecting their samples were excluded [14, 18]. The mean level of albuminuria was categorized according to KDIGO’s clinical guidelines (< 3, 3–30, and > 30 mg/mmol) [17].

All analyses were performed at the Central Laboratory at Levanger Hospital. Non-fasting serum from fresh blood samples was used to analyze serum concentrations of creatinine in HUNT2 by using Hitachi 911 Autoanalyzer (Hitachi, Mito, Japan) with reagents from Boehringer, Mannheim (Mannheim, Germany) and in HUNT3 by an Architect ci8200 Autoanalyzer (Abbott Diagnostics, Langford, Ireland). Creatinine was measured with the Jaffe method but standardized to isotope dilution mass spectroscopy level as previously described [19]. Immunoturbimetric methods were applied to determine urine albumin using antihuman serum albumin, and ACR was calculated in mg/mmol. The suppliers of these methods were DakoAS, Glostrup, Denmark, in HUNT2, and Abbott Laboratories in HUNT3.

### Outcomes

A BSI was defined as the growth of bacteria in one or more blood cultures taken on clinical indication. Isolates solely consisting of microorganisms commonly associated with skin contamination, such as coagulase-negative *Staphylococcus* species*, Corynebacterium* species, and *Propionibacterium* species, were not considered BSI [20]. BSI mortality was defined as death within 30 days after detection of a BSI.

Sepsis was defined retrospectively by using a combination of ICD codes. The ICD by the World Health Organization is the foundation for the identification of health trends and statistics globally and is used in many countries, including Norway, for administrative/economic purposes upon hospital discharge [21]. Clinicians are advised to only use the primary codes for sepsis when the origin of the infection is unknown. A widely used method to overcome this in order to retrospectively correctly identify patients with sepsis is to construct a combination of primary sepsis codes (explicit sepsis) and codes for infection with a known organ focus combined with codes for organ dysfunction (implicit sepsis) [22–25]. In 2020, Rudd et al. [1] published global sepsis incidence and mortality data using this type of approach, and their criteria are used to define sepsis in our analysis. Sepsis mortality was defined as death within 30 days of diagnosis.

For all participants, the unique 11-digit identification number of every Norwegian citizen enabled linkage to the Norwegian population registry to obtain information on the date of emigration and date of death and to electronic health records at all hospitals serving the HUNT cohort area (Levanger Hospital, Namsos Hospital, and the regional tertiary care hospital St Olav’s Hospital). This included information on all positive blood cultures and ICD codes through February 2017.

### Covariates

Diabetes, cardiovascular disease (myocardial infarction or stroke), systolic blood pressure (SBP, mmHg), body mass index (BMI, kg/m^2^), and smoking status (never, former, or current smoker) at baseline were considered relevant covariates in addition to age and sex. Information was missing in less than 1% for all of these variables, and we therefore did not impute missing data.

### Study design and statistical analyses

For each outcome (BSI, BSI mortality, sepsis, and sepsis mortality), we used Cox regression analysis to estimate hazard ratios (HRs) with 95% confidence intervals (CIs) associated with five categories of eGFR with eGFR ≥ 90 ml/min/1.73m^2^ as reference and three categories of ACR with ACR < 3 mg/mmol as reference. We reported unadjusted, age-adjusted, sex-adjusted, and multivariable-adjusted HRs (age at first HUNT participation, sex, diabetes, cardiovascular disease, smoking status, SBP, and BMI). The participants were followed from the day of inclusion to either HUNT2 or HUNT 3 (HUNT 2 if they attended both). In the analysis of the risk of BSI or sepsis, the participants were followed until their first BSI or sepsis, migration out of Nord-Trøndelag county, death, or February 17, 2017, whichever occurred first. In the analysis of mortality, they were followed until migration out of Nord-Trøndelag County, death, or February 17, 2017, whichever occurred first. The proportional hazards assumption was examined using visual inspection of log–log plots and tests of Schoenfeld residuals. All analyses and graphics were carried out using Stata/MP version 16.

## Results

GFR was estimated in a total of 68,438 participants. Their mean (SD) age was 47.7 years (16.6), and 36,286 (53.0%) were female. A total of 9,516 (13.9%) had CKD as defined by eGFR < 60 ml/min/1.73m^2^. Baseline characteristics of the study population are summarized in Table 1 by eGFR categories.

**Table 1:** Baseline characteristics of the study population

Variable	Total population (*n* = 68,438)	eGFR ≥ 90 (*n* = 23,304)	eGFR 60–89 (*n* = 35,618)	eGFR 45–59 (*n* = 7816)	eGFR 30–44 (*n* = 1556)	eGFR < 30 (*n* = 144)
Age, mean (SD)	47.7 (16.6)	33.2 (9.1)	50.8 (12.6)	69.9 (9.4)	77.0 (8.4)	77.3 (9.8)
Female sex, *n* (%)	36,286 (53.0)	9954 (42.7)	19,736 (55.4)	5402 (69.1)	1115 (71.7)	79 (54.8)
Lifestyle factors						
BMI kg/m^2^, mean (SD)	26.4 (4.2)	25.7 (4.1)	26.5 (4.1)	27.7 (4.3)	27.8 (4.4)	28.4 (5.0)
Current smokers, *n* (%)	18,441 (27.4)	6536 (28.4)	10,399 (29.5)	1303 (17.3)	183 (12.6)	20 (14.4)
Former smokers, *n* (%)	18,325 (27.2)	4489 (19.5)	10,875 (30.9)	2501 (33.1)	417 (28.8)	43 (30.1)
Never smokers, *n* (%)	29,220 (43.4)	11,006 (47.8)	13,583 (38.6)	3711 (49.2)	844 (58.3)	76 (54.7)
Alcohol intake units/2 weeks, median (IQR)	2 (0–6)	3 (0–7)	2 (0–5)	0 (0–1)	0 (0–0)	0 (0–0)
Comorbidities						
SBP mmHg, mean (SD)	134. (20.9)	126.4 (14.4)	135.5 (20.1)	152.5 (23.9)	158.7 (26.5)	154.3 (26.8)
Total cholesterol mmol/l, mean (SD)	5.64 (1.19)	5.21 (1.06)	5.75 (1.15)	6.20 (1.32)	6.47 (1.39)	6.48 (1.50)
Diabetes, *n* (%)	2028 (3.0)	72 (0.3)	857 (2.4)	632 (8.1)	221 (14.3)	27 (19.0)
Myocardial infarction, *n* (%)	1809 (2.7)	70 (0.3)	803 (2.4)	585 (8.2)	203 (14.7)	22 (18.5)
Cerebrovascular event, *n* (%)	1169 (1.7)	94 (0.4)	518 (1.5)	388 (5.0)	151 (9.8)	18 (12.7)
Asthma, *n* (%)	6424 (9.4)	2318 (10.0)	3240 (9.1)	711 (9.1)	136 (8.8)	19 (13.2)
Mobility impairment, *n* (%)	22,931 (34.2)	5013 (21.8)	12,401 (35.4)	4367 (59.0)	1049 (72.6)	101 (75.9)

*eGFR* estimated glomerular filtration rate measured in ml/min/1.73m^2^, *BSI* bloodstream infection, *n* number, *BMI* body mass index, *IQR* interquartile range, *SBP* systolic blood pressure

During a median follow-up of 17.4 years, 2362 participants were hospitalized with BSI and 2867 with sepsis. The subjects in the two groups somewhat overlapped, but not completely. Of the participants admitted with sepsis, 49.6% were also registered as having had a BSI, while of the participants never admitted with sepsis, 1.4% had a BSI during the follow-up time. The three predominant bacterial pathogens in BSI were *Escherichia coli, Staphylococcus aureus,* and *Streptococcus pneumoniae* (Supplementary Tables 1 and 3 show detailed lists of microbes for participants with BSI and participants with eGFR < 30 ml/min/1.73m^2^ and BSI, respectively).

**Fig. 1:**
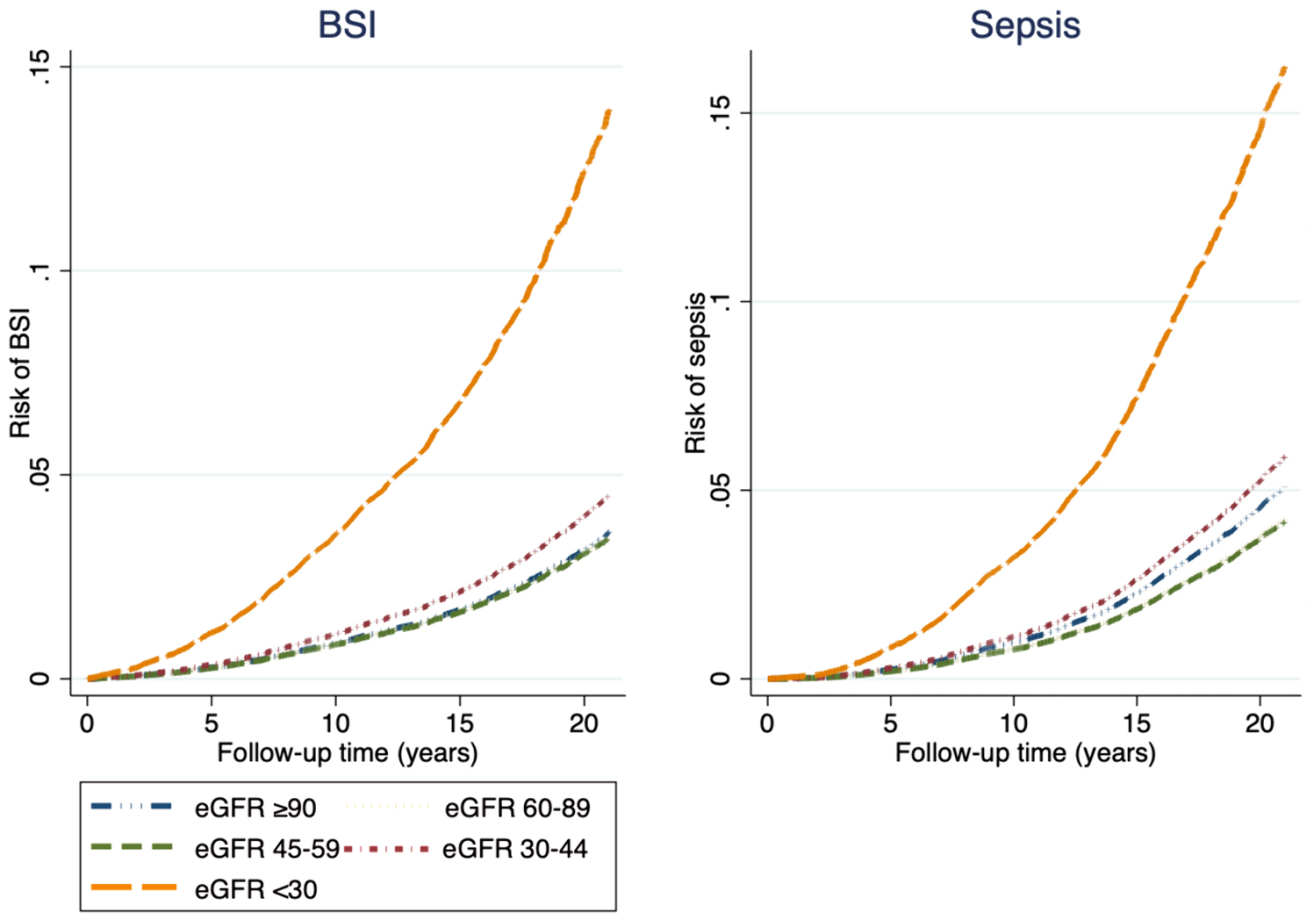
eGFR and risk of BSI and sepsis. Age- and sex-adjusted risk of BSI and sepsis during follow-up by the level of eGFR (ml/min/1.73^2^) upon HUNT entry. *eGFR* estimated glomerular filtration rate, *BSI* bloodstream infection

Lower eGFR was associated with an increased risk of both BSI and sepsis in multivariable-adjusted analyses. There was no increased risk of BSI with eGFR 45–90 ml/min/1.73m^2^, but participants with kidney function below this level had increasing risk. Participants with eGFR 30–44 and < 30 ml/min/1.73m^2^ had HRs of 1.18 (95% CI 0.89–1.55) and 3.35 (95% CI 2.12–5.3), respectively, compared to eGFR ≥ 90 ml/min/1.73m^2^. For sepsis, the HR was 2.94 (95% CI 1.82–4.8) when eGFR < 30 ml/min/1.73m^2^ compared to when eGFR ≥ 90 ml/min/1.73m (Table 2, Fig. 1).

**Table 2:** Estimated glomerular filtration rate (eGFR) and risk of bloodstream infection (BSI) and sepsis

Outcome	eGFR	Person-years at risk	No of cases	Unadjusted			Age- and sex-adjusted			Multiadjusted^a^		
				HR	95% CI	*p*	HR	95% CI	*p*	HR	95% CI	*p*
BSI (*n* = 2362)	≥ 90	344,389	239	Ref	Ref		Ref	Ref		Ref	Ref	
	60–89	583,353	1402	3.35	2.92–3.84	< 0.001	0.98	0.83–1.15	0.77	0.93	0.79–1.08	0.34
	45–59	99,122	573	8.84	7.60–10.28	< 0.001	0.97	0.78–1.18	0.70	0.88	0.71–1.08	0.22
	30–44	13,046	123	17.03	13.69–21.19	< 0,001	1.26	0.96–1.66	0.09	1.18	0.89–1.55	0.24
	< 30	854	25	62.24	41.16–94.10	< 0.001	4.10	2.63–6.40	< 0.001	3.35	2.12–5.27	< 0.001
Sepsis (*n* = 2867)	≥ 90	343,239	321	Ref	Ref		Ref	Ref		Ref	Ref	
	60–89	583,252	1685	2.95	2.61–3.32	< 0.001	0.83	0.71–0.95	< 0.001	0.88	0.76–1.03	0.11
	45–59	99,265	686	8.07	7.07–9.22	< 0.001	0.81	0.68–0.98	0.03	0.88	0.72–1.06	0.19
	30–44	13,106	150	16.97	13.96–20.61	< 0.001	1.16	0.91–1.48	0.23	1.28	0.99–1.67	0.06
	< 30	870	25	53.56	36.61–80.55	< 0.001	3.39	2.20–5.22	< 0.001	2.94	1.82–4.76	< 0.001

*eGFR* estimated glomerular filtration rate measured in ml/min/1.73m^2^, *n* number, *CI* confidence interval, *BSI* bloodstream infection

^a^Diabetes, cardiovascular disease (myocardial infarction and stroke), systolic blood pressure, body mass index, smoking status

Among the 2,362 individuals with a BSI during follow-up, 345 (14.6%) died within 30 days. Of the 2,867 patients admitted with sepsis, 613 (21.4%) died during admission or within 30 days. The risk of dying from either a BSI or during an admission for sepsis showed the same pattern as for acquiring the infections. Participants with eGFR < 30 ml/min/1.73m^2^ had HR 4.2 (95% CI 1.71–10.4) of dying of a BSI compared to eGFR ≥ 90 ml/min/1.73m^2^. The same was seen for sepsis deaths, where the risk of dying was 4.1 times as high in the group with the lowest eGFR compared to participants with normal kidney function (Table 3).

**Table 3:** Estimated glomerular filtration rate (eGFR) and mortality from bloodstream infection (BSI) or sepsis

Outcome	eGFR	Person-years at risk	No of deaths	Unadjusted			Age- and sex-adjusted			Multiadjusted^a^		
				HR	95% CI	*p*	HR	95% CI	*p*	HR	95% CI	*p*
BSI deaths (*n* = 345)	≥ 90	368,207	30	Ref	Ref		Ref	Ref		Ref	Ref	
	60–89	602,504	183	3.62	2.46–5.32	< 0.001	0.67	0.43–1.05	0.08	0.61	0.40–0.96	0.03
	45–59	102,380	101	13.20	8.11–19.84	< 0.001	0.76	0.43–1.32	0.32	0.64	0.36–1.12	0.11
	30–44	13,574	23	27.47	15.91–47.39	< 0.001	1.01	0.50–2.04	0.97	0.92	0.45–1.86	0.81
	< 30	922	8	164.14	74.94–359.53	< 0.001	5.40	2.21–13.18	< 0.001	4.22	1.71–10.41	< 0.001
Sepsis deaths (*n* = 613)	≥ 90	343,239	33	Ref	Ref		Ref	Ref		Ref	Ref	
	60–89	583,252	336	5.74	4.01–8.20	< 0.001	0.83	0.55–1.23	0.36	0.75	0.50–1.13	0.17
	45–59	99,265	180	20.6	14.21–29.9	< 0.001	0.80	0.50–1.28	0.36	0.72	0.45–1.16	0.18
	30–44	13,106	51	55.90	36.01–86.8	< 0.001	1.32	0.76–2.29	0.32	1.20	0.68–2.14	0.52
	< 30	870	13	270.3	141.79–515.27	< 0.001	5.42	2.63–11.18	< 0.001	4.10	1.88–8.92	< 0.001

*eGFR* estimated glomerular filtration rate measured in ml/min/1.73m^2^, *HR* hazard ratio, *CI* confidence interval

^a^Diabetes, cardiovascular disease (myocardial infarction and stroke), systolic blood pressure, body mass index, smoking status

The ACR was measured in a subgroup of 9,699 participants enriched in diabetes mellitus and hypertension. This subgroup had a higher mean age (57.9 years), systolic blood pressure (146.5 mmHg), and diabetes prevalence (15.1%) at baseline. Further characteristics of the study population with data on ACR are summarized in Table 4. The groups of participants with CKD defined by the two different methods (eGFR and ACR) partly overlapped (Supplementary Table 3).

**Table 4:** Baseline characteristics of the population having urine albumin–creatinine ratio (ACR) measured

Variable	Total (*n* = 9699)	ACR < 3 mg/mmol (*n* = 8461)	ACR 3–30 mg/mmol (*n* = 1090)	ACR > 30 mg/mmol (*n* = 148)
Age, mean (SD)	57.9 (15.4)	56.8 (15.4)	65.0 (13.6)	67.2 (13.0)
Female sex, *n* (%)	5170 (53.3)	4603 (54.4)	519 (47.8)	48 (32.4)
Lifestyle factors				
BMI kg/m^2^, mean (SD)	27.9 (4.6)	27.8 (4.5)	28.5 (4.9)	28.6 (4.6)
Current smokers, *n* (%)	2082 (21.9)	1780 (21.4)	260 (24.8)	42 (29.6)
Former smokers, *n* (%)	3201 (33.7)	2748 (33.1)	394 (37.6)	59 (41.6)
Never smokers, *n* (%)	4146 (43.7)	3720 (44.8)	386 (36.8)	40 (28.2)
Alcohol intake units/2 weeks, median (IQR)	0 (0–4)	0 (0–4)	0 (0–4)	0 (0–3)
Comorbidities				
SBP mmHg, mean (SD)	146.5 (23.5)	145.0 (22.9)	155.8 (24.9)	165.0 (24.3)
Total cholesterol mmol/l, mean (SD)	5.78 (1.30)	5.77 (1.28)	5.87 (1.37)	6.26 (1.53)
Diabetes, *n* (%)	1469 (15.1)	1107 (13.1)	313 (28.5)	49 (33.1)
Myocardial infarction, *n* (%)	676 (7.0)	529 (6.3)	123 (11.4)	24 (16.6)
Cerebrovascular incidence, *n* (%)	402 (4.2)	295 (3.5)	83 (7.7)	24 (16.3)
Asthma, *n* (%)	973 (10.0)	833 (9.9)	122 (11.2)	18 (12.2)
Mobility impairment, *n* (%)	4430 (47.1)	3704 (45.0)	632 (61.2)	94 (67.1)

*ACR* albumin–creatinine ratio, *BSI* bloodstream infection, *SD* standard deviation, *n* number, *BMI* body mass index, *IQR* interquartile range, *SBP* systolic blood pressure

**Fig. 2:**
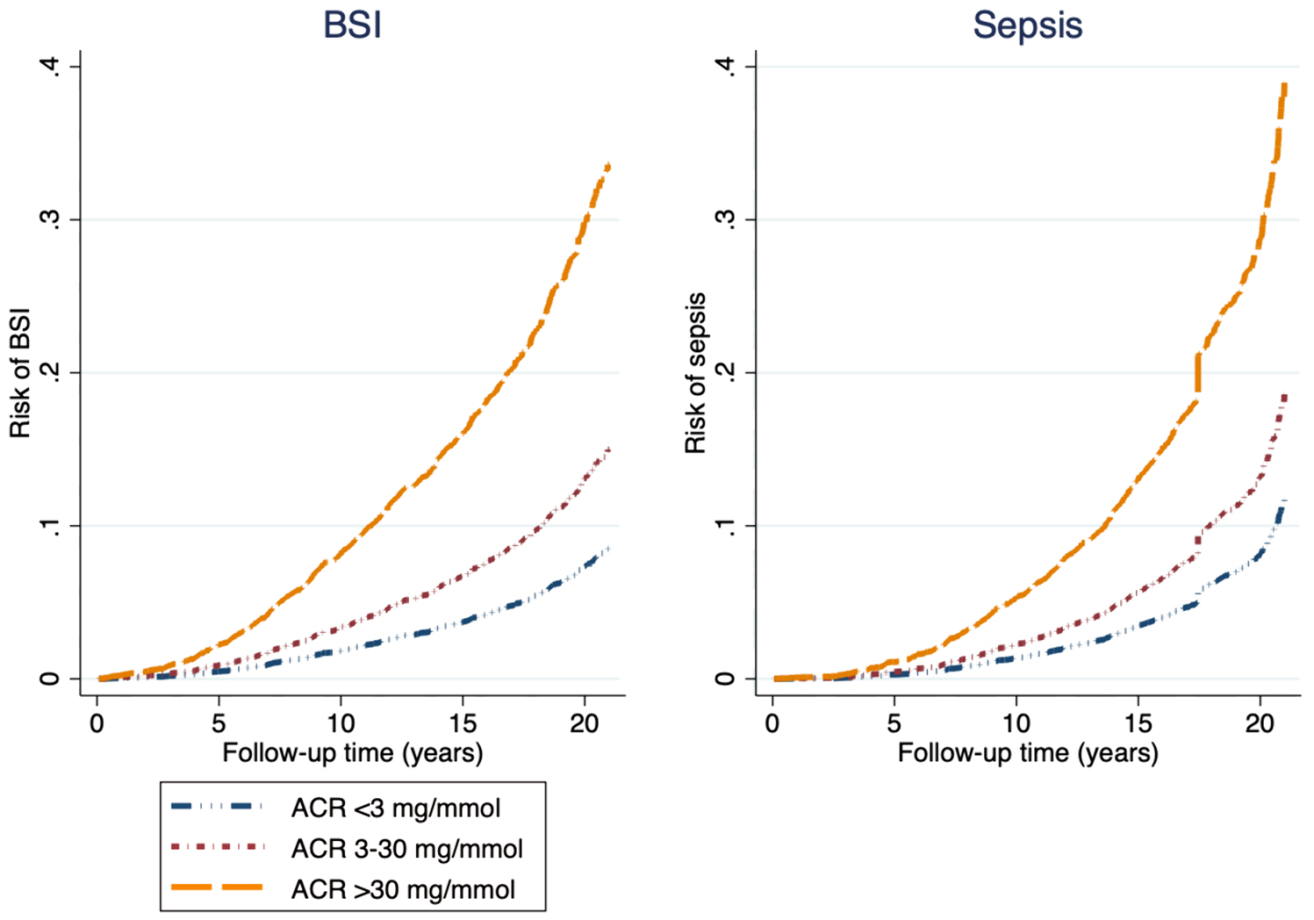
ACR and risk of BSI and sepsis. Age- and sex-adjusted risk of BSI and sepsis during follow-up, by level of ACR (mg/mmol) upon HUNT entry. *ACR* albumin–creatinine ratio, *BSI* bloodstream infection

During a median follow-up time of 17.4 years, 612 of the participants with an ACR measurement had a BSI, and 684 had an admission with sepsis. The severity of increased ACR was associated with an increased risk of both having a BSI and an admission with sepsis. For BSI, participants with ACR 3–30 mg/mmol and > 30 mg/mmol had HRs of 1.80 (95% CI 1.37–2.11) and 3.60 (95% CI 2.30–5.6), respectively. For sepsis, the participants with ACR 3–30 mg/mmol and > 30 mg/mmol had HRs of 1.53 (95% CI 1.24–1.90) and 3.14 (95% CI 1.94–5.1), respectively, compared to normal albumin excretion (Table 5, Fig. 2).

**Table 5:** Albumin–creatinine ratio (ACR) and risk of bloodstream infection (BSI) and sepsis

Outcome	ACR (mg/mmol)	Person-years	No of participants	Unadjusted			Age- and sex-adjusted			Multiadjusted ^a^		
				HR	95% CI	*p*	HR	95% CI	*p*	HR	95% CI	*p*
BSI (*n* = 612)	< 3	131,586	483	Ref	Ref		Ref	Ref		Ref	Ref	
	3–30	12,992	107	2.46	1.99–3.03	< 0.001	1.83	1.48–2.26	< 0.001	1.80	1.37–2.11	< 0.001
	> 30	1301	22	5.60	3.65–8.60	< 0.001	4.61	3.00–7.11	< 0.001	3.60	2.30–5.63	< 0.001
Sepsis (*n* = 684)	< 3	129,027	562	Ref	Ref		Ref	Ref		Ref	Ref	
	3–30	12,830	104	2.15	1.74–2.65	< 0.001	1.66	1.34–2.05	< 0.001	1.53	1.24–1.90	< 0.001
	> 30	1297	18	4.29	2.68–6.86	< 0.001	3.98	2.48–6.38	< 0.001	3.14	1.94–5.06	< 0.001

*ACR* albumin–creatinine ratio, *HR* hazard ratio, *CI* confidence interval, *BSI* bloodstream infection

^a^Diabetes, cardiovascular disease (myocardial infarction and stroke), systolic blood pressure, body mass index, smoking status

Of the 148 participants with ACR > 30 mg/mmol, 12 also had eGFR > 30 ml/min/1.73m^2^. In this subgroup, the risk of BSI and sepsis was very high (Supplementary Table 4); however, the numbers of events were small; six (50.0%) got BSI and two (16.7%) got sepsis.

In the ACR cohort, 102 people died of a BSI, and 159 died of sepsis. Participants with ACR 3–30 mg/mmol and > 30 mg/mmol had HRs of 1.28 (95% CI 0.70–2.30) and 2.67 (95% CI 0.82–8.73), respectively, of dying of a BSI compared to normal albumin excretion. The same pattern was observed for sepsis. This suggests a higher risk of death from BSI and sepsis in patients with high ACR; however, the CIs here were too wide to draw conclusions due to the low number of deaths in the most severe groups. The findings are summarized in Table 6.

**Table 6:** Albumin–creatinine ratio (ACR) and risk of dying of a bloodstream infection (BSI) or sepsis

Outcome	ACR (mg/mmol)	Person-years	No of deaths	Unadjusted			Age- and sex-adjusted			Multiadjusted^a^		
				HR	95% CI	*p*	HR	95% CI	*p*	HR	95% CI	*p*
Death from BSI (*n* = 102)	< 3	132,984	81	Ref	Ref		Ref	Ref		Ref	Ref	
	3–30	13,289	18	2.44	1.46–4.11	< 0.001	1.61	0.96–2.70	0.07	1.28	0.70–2.30	0.40
	> 30	1363	3	4.31	1.36–13.7	< 0.001	3.26	1.02–10.4	0.05	2.67	0.82–8.73	0.14
Death from sepsis (*n* = 169)	< 3	128,377	140	Ref	Ref		Ref	Ref		Ref	Ref	
	3–30	12,757	25	1.98	1.29–3.03	< 0.001	1.24	0.80–1.91	0.33	1.11	0.71–1.73	0.65
	> 30	1280	4	3.49	1.29–9.43	< 0.001	2.59	0.95–7.06	0.06	2.16	0.78–5.96	0.13

*ACR* albumin–creatinine ratio, *HR* hazard ratio, *CI* confidence interval, *BSI* bloodstream infection

^a^Diabetes, cardiovascular disease (myocardial infarction and stroke), systolic blood pressure, body mass index, smoking status

## Discussion

To the best of our knowledge, we present the largest population-based study with long-term follow-up showing a strong association between CKD and the risk of both admission with and death from BSI and sepsis, independent of the considered confounders. The risk increased with both lower eGFR and higher ACR levels, the two components of a CKD diagnosis.

Some previous studies have also suggested a relationship between CKD and infection. In 2020, Dagasso et al. [7] showed an association between reduced eGFR and community-onset BSI in a Canadian population cohort; however, they did not include ACR or sepsis as a clinical outcome. Wang et al. [5] and James et al. [8] both conducted studies including both eGFR and ACR; however, these were population-based studies, and their follow-up times were considerably shorter. Moreover, Wang et al. only had deaths as an outcome. Other studies have examined CKD and its associated risk with pneumonia and other infections in general [10, 26–28]. Overall, direct comparison with previous work is difficult, as this relationship has been examined in either smaller cohorts, cohorts with shorter follow-up times, or selected groups of patients [5–10, 23].

The link between CKD and severe infections has many potential explanations, and it is likely to be multifactorial. Several consequences of CKD, such as malnutrition, chronic inflammation, uremia, metabolic abnormalities, and abnormalities in neutrophil and lymphocyte function, could contribute to increased infection risk in these patients [28]. Dialysis therapy itself is certainly a known risk factor for invasive infections [28–30], particularly for BSI, both in the setting of chronic renal replacement therapy and when in need of acute dialysis. The proportion of participants in need of dialysis was probably very low in our cohort; however, it was unfortunately not possible to identify them. In addition to acute dialysis, other types of instrumentation such as insertion of central venous lines, peripheral venous lines, lines for invasive monitoring, and urinary bladder catheters could play an important role. These procedures could be more frequently performed in patients with CKD, especially in the setting of acute kidney injury, which has been shown to rise in the setting of decreased eGFR [31]. Unfortunately, our data did not allow us to assess this further; however, we did see that the rate of *Enterococci,* a bacteria highly related to hospital infections and instrumentation [32], was higher in BSI in the subgroup with eGFR < 30 ml/min/1.73m^2^ compared to the total rate (Supplementary Tables 1 and 2). The same is the case with *Staphylococcus aureus*, which was also slightly higher in this subgroup.

Reduced eGFR resulting in uremia has long been considered to give a state of immune hyporesponsiveness, related to the uremia toxin’s ability to impair the functions of T lymphocytes and antigen-presenting cells, changing the T-helper cells toward T-helper-1-function and thus have important roles in both cellular and humoral immunity [33]. Another hypothesis suggests that reduced eGFR and hence a reduced number of nephrons will lower the production of uromodulin, a protein exclusively produced by the kidney, which has an increasing focus on its clinical relevance as a protective factor against urinary tract infections [34–36].

The role of albuminuria as an independent risk factor for sepsis probably has a separate mechanism and is less clear. There is, however, growing evidence of albuminuria’s role as a general marker of inflammation, shown to be associated with several different inflammatory diseases [37–39]. Endothelial dysfunction with increased permeability is thought to be an important mechanism, and this is again closely linked to sepsis [40, 41], independent of eGFR. Heavy proteinuria giving hypogammaglobulinemia and increased risk of especially respiratory infections could also play a role, which has earlier been shown to be a consequence of nephrotic syndrome [14].

### Strengths and limitations

The major strengths of our study include its large size, the population-based design and long-term follow-up of a stable population, and the available information on potential confounding factors. The involvement of only two local hospitals with a shared laboratory and linkage to complete microbiological records also adds strength to our study.

The long-term follow-up is a strength but also a possible limitation, as changes within a healthcare system will occur over time, in addition to possible changes in health-seeking behavior in an increasingly informed cohort. We must also consider the possibility of selection bias. A total of 54–69% of the invited population participated, and although this is a high participation rate for a health survey, high-risk groups for both chronic disease and infection could be underrepresented. As always in observational studies, there is a possibility of unknown confounding factors not thought of or corrected for. The need for dialysis therapy, in both the chronic setting and acute setting, is an important factor that we were not able to assess, in addition to other types of acute procedures such as insertion of central venous lines. Another important possible limitation is the use of ICD coding to retrospectively define sepsis. There is ongoing work and discussion regarding this, and no method of retrospective sepsis definition is without potential fallbacks. Also, the use of ICD coding did not allow us to assess the timing of the different diagnoses during the same admission, such as the timing and relationship between an acute kidney injury and infection.

In conclusion, in this large prospective population-based study, we show that CKD estimated by low eGFR and high ACR in urine increases the risk of both admission and death from BSI and sepsis. Future studies should explore whether preventing, identifying, and correctly treating CKD could have an impact on the risk of invasive infections and death from them.

## Supplementary Information

Below is the link to the electronic supplementary material.

Supplementary file1 (DOCX 28 KB)

## Data Availability

The data that support the findings of this study can be made available from the Trøndelag Health Study (HUNT) (kontakt@hunt.ntnu.no), St Olav’s Hospital and Levanger Hospital. Restrictions apply to the availability of these data, which were used under license for the current study.
