# Supplementary material for: Chronic kidney disease and risk of bloodstream infections and sepsis: a 17-year follow-up of the population-based Trøndelag Health Study in Norway
Source: Infection. 2024 Apr 29;52(5):1983–93. doi: 10.1007/s15010-024-02265-2 (PMC11499395; doi:10.1007/s15010-024-02265-2)
Supplement: Supplementary file 1 — Supplementary file1 (DOCX 28 KB) [file 15010_2024_2265_MOESM1_ESM.docx]

# Chronic kidney disease and risk of bloodstream infections and sepsis: A 17-year follow-up of the population-based Trøndelag Health Study in Norway.

Kristin Vardheim Liyanarachi, MD^1,2^, Randi Marie Mohus, MD, PhD ^1,3^*, Tormod Rogne, MD, PhD ^1,4^*, Lise Tuset Gustad, PhD ^1,5,6^, Bjørn Olav Åsvold, MD, PhD ^7,8,9^, Solfrid Romundstad, MD, PhD ^6,10,^ Erik Solligård, MD, PhD^1,11^, Stein Hallan, MD, PhD ^10,12^, Jan Kristian Damås, MD, PhD^1, 2, 13^

*: Equally contributed.

1. Mid-Norway Center for Sepsis Research, Department of Circulation and Medical Imaging, NTNU, Norwegian University of Science and Technology, Trondheim, Norway
2. Department of Infectious Diseases, St. Olavs Hospital, Trondheim University Hospital, Trondheim, Norway
3. Clinic of Anaesthesia and Intensive Care, St. Olavs Hospital, Trondheim University Hospital, Trondheim, Norway
4. Yale Department of Chronic Disease Epidemiology and Center for Perinatal, Pediatric and Environmental Epidemiology, Yale School of Public Health, New Haven, CT, USA
5. Faculty of Nursing and Health Sciences, Nord University, Levanger, Norway
6. Department of Internal Medicine, Levanger Hospital, Nord-Trøndelag Hospital Trust, Levanger, Norway.
7. K.G. Jebsen Center for Genetic Epidemiology, Department of Public Health and Nursing, NTNU, Norwegian University of Science and Technology, Trondheim, Norway
8. Department of Endocrinology, Clinic of Medicine, St. Olavs Hospital, Trondheim University Hospital, Trondheim, Norway
9. HUNT Research Center, Department of Public Health and Nursing, NTNU, Norwegian University of Science and Technology, Levanger, Norway
10. Department of Clinical and Molecular Medicine, NTNU, Norwegian University of Science and Technology, Trondheim, Norway
11. Helse Møre og Romsdal Hospital Trust, Ålesund, Norway
12. Department of Nephrology, St Olavs Hospital, Trondheim University Hospital, Trondheim, Norway.
13. Centre of Molecular Inflammation Research, Department of Clinical and Molecular Medicine, NTNU, Norwegian University of Science and Technology, Trondheim, Norway

Corresponding author: Kristin Vardheim Liyanarachi

Email: kristin.v.liyanarachi@ntnu.no

## **Supplementary Table 1**: List of microbes identified in the 2,362 participants with BSI.

| Microbe | n (%) |
| --- | --- |
| Gram positive cocci |  |
| *Staphylococcus aureus* | 334 (14.1) |
| *Streptococcus pneumoniae* | 255 (10.8) |
| *Enterococcus sp.* | 133 (5.6) |
| *Betahaemolytic streptococci* | 131 (5.5) |
| *Alphahaemolytic streptococci* | 93 (3.9) |
| *Other* | 17 (0.7) |
| *Aerococcus urinae* | 7 (0.3) |
| Gram negative cocci |  |
| *Neisseria sp.* | 6 (0.3) |
| Gram positive rods |  |
| *Listeria sp.* | 13 (0.6) |
| *Clostridium sp.* | 13 (0.6) |
| *Unidentified* | 12 (0.5) |
| Gram negative rods |  |
| *Escherichia coli* | 653 (27.6) |
| *Unidentified* | 284 (12.0) |
| *Klebsiella sp.* | 129 (5.5) |
| *Pseudomonas sp.* | 58 (2.5) |
| *Bacteroides sp.* | 45 (1.9) |
| *Other* | 38 (1.6) |
| *Proteus sp.* | 36 (1.5) |
| *Other anaerob* | 34 (1.4) |
| *Enterobacter sp.* | 31 (1.3) |
| *Haemophilus sp.* | 18 (0.8) |
| *Serratia sp.* | 14 (0.6) |
| *Salmonella sp.* | 8 (0.3) |

Abbreviations: n, number; sp, species

## **Supplementary Table 2:** List of microbes identified in the 25 participants with eGFR < 30 ml/min/1.73m^2^ and BSI.

| Microbe | n (%) |
| --- | --- |
| Gram positive cocci |  |
| *Staphylococcus aureus* | 4 (16.0) |
| *Enterococcus sp.* | 4 (16.0) |
| *Betahaemolytic streptococci* | 2 (8.0) |
| *Streptococcus pneumoniae* | 1 (4.0) |
| Gram negative cocci |  |
| - |  |
| Gram positive rods |  |
| *Listeria sp.* | 1 (4.0) |
| Gram negative rods |  |
| *Escherichia coli* | 4 (16.0) |
| *Unidentified* | 4 (16.0) |
| *Klebsiella sp.* | 3 (12.0) |
| *Other* | 2 (8.0) |

Abbreviations: n, number; sp, species

## **Supplementary table 3:** Proportion of overlap between the two definitions of chronic kidney disease (CKD).

|  | ACR < 3 (n=8,461) | ACR 3-30 (n=1,090) | ACR >30 (n=148) |
| --- | --- | --- | --- |
| eGFR ≥90, n (%) | 1,331 (15.7) | 66 (6.1) | 7 (4.7) |
| eGFR 60-89, n (%) | 4,726 (55.9) | 506 (46.4) | 44 (29.7) |
| eGFR 45-59, n (%) | 1,968 (23.3) | 372 (34.1) | 43 (29.1) |
| eGFR 30-44, n (%) | 414 (4.9) | 129 (11.8) | 42 (28.4) |
| eGFR < 30, n (%) | 22 (0.3) | 17 (1.6) | 12 (8.1) |

Abbreviations: ACR, albumin creatinine ratio; eGFR, estimated glomerular filtration rate; n, number

|  |  |  |  | Unadjusted | | | Age- and sex-adjusted | | | Multiadjusted ^a^ | | |
| --- | --- | --- | --- | --- | --- | --- | --- | --- | --- | --- | --- | --- |
| Outcome | ACR (mg/mmol) | Person-years | No of participants | HR | 95% CI | p | HR | 95% CI | p | HR | 95% CI | p |
| BSI (n=612) | <3 | 131,586 | 483 | Ref. | Ref. |  | Ref. | Ref. |  | Ref. | Ref. |  |
|  | 3-30 | 12,992 | 107 | 2.46 | 1.99-3.03 | <0.001 | 1.83 | 1.48-2.26 | <0.001 | 1.80 | 1.37-2.11 | <0.001 |
|  | >30 | 1,301 | 22 | 5.60 | 3.65-8.60 | <0.001 | 4.61 | 3.00-7.11 | <0.001 | 3.60 | 2.30-5.63 | <0.001 |
|  | >30 and eGFR < 30 | 56 | 6 | 56.3 | 25.0-126.9 | <0.001 | 28.2 | 12.4-64.1 | <0.001 | 27.6 | 11.9-63.6 | <0.001 |
| Sepsis (n=684) | <3 | 129,027 | 562 | Ref. | Ref. |  | Ref. | Ref. |  | Ref. | Ref. |  |
|  | 3-30 | 12,830 | 104 | 2.15 | 1.74-2.65 | <0.001 | 1.66 | 1.34-2.05 | <0.001 | 1.53 | 1.24-1.90 | <0.001 |
|  | >30 | 1,297 | 18 | 4.29 | 2.68-6.86 | <0.001 | 3.98 | 2.48-6.38 | <0.001 | 3.14 | 1.94-5.06 | <0.001 |
|  | >30 and eGFR < 30 | 56 | 2 | 26.7 | 6.62-107.7 | <0.001 | 14.0 | 3.45-56.8 | <0.001 | 12.3 | 3.00-50.6 | 0.001 |

## **Supplementary Table 4**: Albumin-creatinine ratio (ACR) and risk of bloodstream infection (BSI), including combined group with both ACR> 30 mg/mmol and eGFR < 30 ml/min/1.73m^2^

^a^ Diabetes, cardiovascular disease (myocardial infarction and stroke), systolic blood pressure, body mass index, smoking status

Abbreviations: ACR, albumin creatinine ratio; HR, hazard ratio; CI, confidence interval; BSI, bloodstream infection
